# Validierung des Düsseldorfer Screeningtools: ein traitbasierter Ansatz zur Erfassung der psychischen Belastung von Krebspatienten

**DOI:** 10.1007/s00106-020-00980-4

**Published:** 2020-12-18

**Authors:** Franziska Sisolefsky, Madiha Rana, Majeed Rana, Philipp Y. Herzberg

**Affiliations:** 1 Professur für Persönlichkeitspsychologie und Psychologische Diagnostik, Helmut-Schmidt-Universität/Universität der Bundeswehr Hamburg, Holstenhofweg 85, 22043 Hamburg, Deutschland; 2 Professur für Angewandte Psychologie, Europäische Fernhochschule Hamburg, Doberaner Weg 20, 22143 Hamburg, Deutschland; 3 Klinik für Mund‑, Kiefer und plastische Gesichtschirurgie, Universitätsklinikum Düsseldorf, Moorenstraße 5, 40225 Düsseldorf, Deutschland

**Keywords:** Psychoonkologie, Ressourcen und Risikofaktoren, Unterstützungsbedürfnisse, Mundkrebs, Psycho-Oncology, Resources and risk factors, Supportive care needs, Oral cancer

## Abstract

**Hintergrund:**

Screening von psychischen Belastungen während einer Krebserkrankung ist notwendig, um gezielt die Patienten herauszufiltern, die psychologische Unterstützung benötigen. Derzeit geschieht dies überwiegend über die Abfrage akuter Probleme. Stabile interne und externe Risikofaktoren und präventiv wirkende Merkmale bleiben weitestgehend unberücksichtigt. Das neu entwickelte Düsseldorfer Screeningtool (DST) erfasst psychische Belastung mithilfe stabiler Traits, unter Berücksichtigung der sozialen Unterstützung und des Krankheitsverarbeitungsstils. Zielsetzung ist eine Validierung des DST anhand des Distress-Thermometers (DT) sowie der Psychoonkologischen Basisdokumentation (PO-Bado).

**Methodik:**

Untersucht wurden 126 Patienten mit Plattenepithelkarzinomen im Bereich Hals und Kopf. Zur Festlegung des Cut-Off-Wertes wurden ROC Kurven (Receiver-Operating-Characteristics) berechnet. Als Maß für die Güte werden Area under Curve Werte (AUC) angegeben. Sensitivität und Spezifität wurden für den jeweils als Goldstandard genutzten Fragebogen festgelegt.

**Ergebnisse:**

Die Diskriminationsfähigkeit des DST ist sowohl im Vergleich mit dem DT als auch mit der PO-Bado mit AUC-Werten von 0,62 bis 0,80 als gut zu bewerten. Bei einem Cut-Off-Wert des DT von 5 kann eine Sensitivität von 84,2 % bei gleichzeitiger Spezifität von 37,0 % angegeben werden. Im Vergleich zur PO-Bado kann ein Sensitivitätswert von 92,9 % bei gleichzeitiger Spezifität von 43,6 % angegeben werden.

**Schlussfolgerung:**

Die Ergebnisse zeigen, dass auch ein traitbasierter Ansatz zur Erfassung von psychischer Belastung zielführend und sicher die Belastungssituation von Krebspatienten aufzeigen kann, sodass sich hier ein neuer Ansatz des Screenings in der Psychoonkologie herauskristallisiert hat.

**Zusatzmaterial online:**

Die Online-Version dieses Beitrags (10.1007/s00106-020-00980-4) enthält eine Tabelle zur Stichprobenbeschreibung sowie erläuternde Skalen. Beitrag und Zusatzmaterial stehen Ihnen auf www.springermedizin.de zur Verfügung. Bitte geben Sie dort den Beitragstitel in die Suche ein, das Zusatzmaterial finden Sie beim Beitrag unter „Ergänzende Inhalte“.

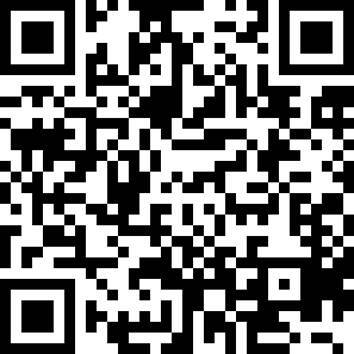

## Abstract (en)

**Background:**

Psycho-oncological screening is a useful tool to ascertain whether patients need special support. Currently, patients are asked mainly about their acute problems. Stable internal and external risk factors and preventive patient characteristics are largely disregarded. The newly developed Düsseldorf Screening Tool (DST) captures distress through stable traits, social support, and the patient’s coping style. The aim of this study was to validate the DST using the Distress-Thermometer (DT) and Basic Documentation for Psycho-Oncology (PO-Bado).

**Methods:**

A total of 126 patients with head and neck squamous cell carcinoma were investigated. To determine the cut-off value, receiver operating curves (ROC) curves were calculated. As a measure of quality, area under the curve (AUC) values are given. Sensitivity and specificity were established for each gold standard questionnaire.

**Results:**

Compared to the DT and PO-Bado, the discrimination ability of the DST is good, with AUC values of 0.62 to 0.80. With a DT cut-off value of 5, a sensitivity of 84.2% with a specificity of 37.0% can be stated. Compared to PO-Bado, a sensitivity value of 92.9% with a specificity of 43.6% can be reported.

**Conclusion:**

The results show that a trait-based approach to identify patients’ level of psychological distress can purposefully and safely assess cancer patients’ stress level. A new approach to screening in psycho-oncology has thus emerged.

Die Notwendigkeit psychoonkologischer Unterstützung während einer Krebserkrankung ist unstrittig. Die Mittel hierfür sind in Kliniken oft begrenzt. Daher ist es wichtig, schnell und einfach zu entscheiden, welche Patienten besonderen Unterstützungsbedarf haben und welche die Erkrankung unter Nutzung persönlicher Ressourcen verarbeiten können. Um dies zu erkennen, werden psychoonkologische Screenings genutzt. Der wissenschaftliche und praktische Mehrwert des im Folgenden vorgestellten Screening-Instruments besteht in der ergänzenden Diagnostik von persönlichen Risikofaktoren und Ressourcen der Patienten.

## Forschungskontext und Fragestellung

Zahlreiche epidemiologische Studien und Metaanalysen zur psychischen Komorbidität bei einer Krebserkrankung zeigen, dass durchschnittlich 32 % aller Krebspatienten eine psychische Störung aufweisen. Am häufigsten kommen Anpassungsstörungen, Angststörungen und Depressionen vor. Auch Fatigue (56 %), Schlafprobleme (51 %), Sorgen und Ängste (47 %) sind keine Seltenheit [12]. Um mit den unterschiedlichen Kapazitäten der psychoonkologischen Betreuungsmöglichkeiten auf diese Komorbiditäten gezielt einzugehen, ist es notwendig, Patientenbedürfnisse und den akuten Behandlungsbedarf priorisieren zu können. In den S3-Leitlinien zur Psychoonkologischen Diagnostik, Beratung und Behandlung von erwachsenen Krebspatienten ist hierzu das psychologische Screening bereits seit 2014 als handlungsleitend vorgegeben [3]. Nicht nur entsprechend dieser Maßgabe ist der standardisierte Einsatz von Screenings für eine valide Beurteilung der Patienten unabdingbar [13]. Einzig die gezielte Abfrage der individuellen Belastung jedes Patienten kann die – durch die Leitlinien außerdem geforderte – konsequente psychoonkologische Unterstützung sicherstellen. Hierbei sind die Art der Tumorerkrankung, die akute Belastung und der subjektiv wahrgenommene Unterstützungsbedarf des Patienten zu berücksichtigen.

Weis et al. fassen die sich daraus ableitenden Anforderungen an Screenings wie folgt zusammen:

- schnell durchführbar,
- verständlich für den Patienten,
- praktikable und einfache Durchführung,
- schnelle und einfache (automatisierte) Auswertung [24].

Essenziell sind demnach betriebswirtschaftliche Effizienz und diagnostische Präzision. Das Verfahren soll zudem von allen Personen im Behandlungsteam eingesetzt werden [9].

Etablierte Verfahren ermitteln jedoch vorrangig das aktuelle Befinden der Patienten in einem vorgebebenen zeitlichen Bezugsrahmen (bspw. den letzten drei Tagen). Individuelle Ressourcen oder Risikofaktoren bleiben unberücksichtigt. Somit ist der Fokus überwiegend problemorientiert und gegenwartsbezogen. Strittmacher et al. haben bereits 2002 darauf aufmerksam gemacht, dass Screenings langfristige Risikofaktoren ebenso wie protektive Faktoren ermitteln sollten, um positive Ressourcen im Rahmen psychoonkologischer Interventionen gezielt zu stärken [22]. Zahlreiche Studien betonen darüber hinaus die Notwendigkeit, den Wunsch nach Unterstützung zu erfragen [2, 24]. Dieser Aspekt kommt durch ergänzende Fragen in neueren Studien bereits zur Anwendung [6]. Die Abfrage nach destabilisierenden Risikofaktoren und möglichen vorhandenen Ressourcen ist derzeit jedoch in keinem Screening etabliert. Somit bleiben wertvolle Erkenntnisse unberücksichtigt.

Zu den wesentlichen Prädiktoren gehört die Persönlichkeit der Patienten. So fanden Warbah et al. signifikante Zusammenhänge zwischen Neurotizismus und Distress [23]. Rana et al. hingegen konnten Persönlichkeitseigenschaften wie Optimismus, Lebenszufriedenheit oder Gewissenhaftigkeit als Ressourcen herausarbeiten, die einen positiven Einfluss auf die Krankheitsverarbeitung hatten und somit die Belastung der Patienten senkten [17].

Ebenfalls gut erforscht ist die positive Wirkung sozialer Unterstützung [1]. Insbesondere das Verhältnis zum Partner gilt als guter Prädiktor für eine hohe Lebensqualität und ein niedriges Belastungsniveau. Eine funktionierende dyadische Copingstrategie führt demnach zu einer klinisch relevanten Verbesserung der Lebensqualität und liefert einen substanziellen Beitrag zur Deckung des jeweiligen Unterstützungsbedarfs. Lebenspartner können somit die knappen Ressourcen einer professionellen Unterstützung entlasten [4].

Um diese Anforderungen an moderne und leistungsfähige Screenings zu erfüllen, haben Sisolefsky et al. das Düsseldorfer Screeningtool (DST) entwickelt [21]. Dieses misst Distress anhand stabiler Eigenschaften der Persönlichkeit. Persönlichkeit gilt als langfristiger Einflussfaktor auf die Krankheitsverarbeitung und damit auch die psychische Belastung über den gesamten Zeitraum der Erkrankungsdauer [17, 21]. Zudem erfasst das DST den Krankheitsverarbeitungsstil sowie die divergierende soziale Unterstützung der Patienten. Anders als derzeit genutzte Fragebögen können Risikofaktoren der Patienten identifiziert und zur Einschätzung der Resilienz genutzt werden. Auf der Grundlage einer qualitativen Interviewstudie wurden vorab durch Ärzte aus dem Bereich der Onkologie sowie durch Krebspatienten die Eigenschaften identifiziert, die sich in der Praxis als klinisch und psychologisch relevant darstellen. Die Augenschein- und externe Validität ist demnach gewährleistet [18]. Das DST wurde zum Einsatz in der Primärtherapie entwickelt. Durch den Rückgriff auf stabile Ressourcen und Risikofaktoren der Patienten kann eine Anwendung jedoch auch in nachfolgenden Behandlungen zur Anwendung kommen.

Ziel dieser Studie ist es, die Konstruktvalidität des DST anhand des Distress-Thermometers (DT) und der psychoonkologischen Basisdokumentation (PO-Bado) nachzuweisen. Zusätzlich soll ein für die Praxis sinnvoller Cut-off-Wert ermittelt sowie Sensitivität und Spezifität bestimmt werden.

## Methodik

### Studiendesign und Stichprobe

Die Stichprobengröße wurde mittels dem Programm G*Power a posteriori überprüft und gilt für alle durchgeführten statistischen Analysen als ausreichend groß [5]. Intention war die Generierung einer aussagekräftigen Stichprobe, bei gleichzeitiger Berücksichtigung der Ökonomie und Vermeidung einer unnötigen Belastung der Patienten durch psychoonkologische Forschungsanliegen. Die Power für Korrelationen und Mittelwertvergleiche ist bei *p* < 0,05 jeweils >0,90. Für die ROC-Analysen wurde aus Tab. 3 der niedrigste AUC-Wert von 0,64 eingesetzt. Bei *n* = 126 konnte eine Power von 0,88 ermittelt werden. Daraus ergibt sich für die anderen AUC-Werte eine Power von >0,90.

In dieser querschnittlichen multizentrischen Untersuchung wurden *n* = 176 Patienten mit Plattenepithelkarzinomen (PEC) im Bereich des Halses und Kopfes aus den Universitätskliniken Hannover und Düsseldorf im Rahmen der Primärtherapie für eine Teilnahme befragt. Zielsetzung war der retrospektive Blick auf die psychische Belastung der Patienten vom Zeitpunkt der Diagnose bis zum jetzigen Stand der Behandlung. Die Daten wurden in den Jahren 2016 bis 2020 erhoben. Ethikvoten beider Universitätskliniken liegen vor (Hannover: 2452-2014, Düsseldorf: 2017094446). Jeder Patient wurde vor Aushändigung der Fragebögen umfassend aufgeklärt. Knapp 22 % der Patienten verweigerten eine Teilnahme aus Zeitgründen, 4 % fanden die gestellten Fragen zu intim. Aufgrund anderer Diagnosen wurden 3 Patienten nicht in die Studie aufgenommen. Einschlusskriterien waren: Volljährigkeit, Erst- oder Nebendiagnose Plattenepithelkarzinom, in Behandlung in Primärtherapie und die schriftliche Einwilligungserklärung zur Teilnahme an der Studie. Ausschlusskriterien waren: Multimorbidität, geistige Behinderung, Vormundschaft und der Konsum illegaler Substanzen. Daten hierzu wurden sowohl durch die mündliche Befragung des Patienten sowie im Zweifel durch Rückgriff auf die Patientenakte gewonnen. Für die Datenanalyse wurden somit *n* = 126 Datensätze berücksichtigt. Bis dato befassen sich im Vergleich zu anderen onkologischen Krankheitsbildern wenige Studien mit dieser Patientenklientel, obwohl die Inzidenz stark ansteigend ist und diese Tumoren hinsichtlich Neuerkrankungen mittlerweile an 7. Stelle weltweit stehen [19]. Diesem Umstand wird mit der Wahl dieser Patientenklientel Rechnung getragen.

Die Teilnehmer sind mehrheitlich männlich (*n* = 76; 60 %). Das Durchschnittsalter beträgt 66,46 Jahre (*SD* = 11,45, Range 28–89), wobei die Frauen mit durchschnittlich 68,73 Jahren (*SD* = 12,30) älter sind als die Männer (64,97; *SD* = 10,68). Die Mehrzahl der Patienten (56 %) sind verheiratet und haben Kinder (79 %). Am häufigsten weisen die Patienten Tumoren auf, die sich überlappend über mehrere Teilbereiche (27 %) erstrecken. Tumoren an Zunge (18 %) und Mundboden (18 %) sind ebenfalls häufig. Eine tabellarische Stichprobenbeschreibung findet sich als Supplementary Material anhängend.

### Instrumente

Das DST wurde in einem dreischrittigen Verfahren entwickelt. Hierzu wurden zunächst in zwei qualitativen Interviewstudien mit Ärzten und betroffenen Patienten die Faktoren herausgearbeitet, die aus Sicht beider Gruppen einen Einfluss auf die psychische Belastung sowie die Lebensqualität der Patienten haben [18]. Im nächsten Schritt wurden diese Ergebnisse zusammengetragen und im Rahmen einer weiteren Vorstudie mittels Thinking-Aloud-Methode 70 Patienten mit PEC vorgelegt. Anschließend wurden mittels Faktoranalyse Items mit unzureichender Faktorladung sowie geringer Item-Skalenkorrelation und bei verringernden Auswirkungen auf die Skalenhomogenität ausselektiert. Hieraus entstand die für diese Studie genutzte Version des DST [21]. Eine Übersicht aller Items und der dazugehörigen Kennwerte findet sich ebenfalls im Supplementary Material.

Das DST erfasst Distress infolge einer Krebserkrankung. Es umfasst zwei Skalen sowie vier ergänzende Items. In Skala 1 erfassen 20 Items den subjektiv empfundenen Distress, welcher als persönliches Ressourcendefizit langfristig wirkender Präventivfaktoren operationalisiert wurde. Diese 20 Items bilden den Kern des DST. Die Auswertung erfolgt mittels Summenscore: Je höher der Score, desto ausgeprägter werden Risikofaktoren bewertet, die die Entstehung von Distress begünstigen. Skala 2 erfasst mit zehn weiteren Items positive Ressourcen der Patienten. Hiermit wird es den Behandelnden ermöglicht, psychoonkologische Interventionspotenziale zu erkennen und in die Therapieplanung miteinzubeziehen. Skala 2 unterstützt somit qualitativ die Entwicklung einer Betreuungsstrategie zur Reduzierung von Distress. Sie wurde daher im Rahmen dieser Validierungsstudie zur Erhebung eines Cut-off-Werts nicht mitbetrachtet. Die Tab. 1 enthält einige Beispielitems der beiden Skalen. Der subjektive Unterstützungsbedarf der Patienten wird mit vier weiteren Items erfasst, wobei drei Items nach der Art der gewünschten Unterstützung fragen (Seelsorge, psychologische Beratung oder Selbsthilfegruppe). Ein Item erfasst, ob die Patienten für die Verarbeitung ihrer Erkrankung Hilfe wünschen. Das DST repräsentiert hiermit die Schlüsseleigenschaften der verschiedenen Einflussfaktoren, die in den vorangegangenen Studien identifiziert wurden, mit einer oder mehreren Fragen. Das DST ist auf eine vierstufigen Likert-Skala mit den Polen *stimmt gar nicht* bis *stimmt voll und ganz* skaliert, um die Tendenz zur Mitte und somit die fehlerhafte Begrenzung der Primärvarianz zu verhindern. Zum Ausfüllen des DST benötigen die Patienten maximal fünf Minuten.

**Table 1:** 

Skala	Item
Skala 1 Ressourcendefizite	Ich fühle mich häufig allein gelassen
	Nach einem Rückschlag erhole ich mich nur schwer
Skala 2 positive Ressourcen	Ich kämpfe entschlossen gegen meine Erkrankung an
	Ich genieße jeden Moment, in dem es mir gut geht

Neben dem DST wurden zur Prüfung der Konstruktvalidität, der Sensitivität und Spezifität zwei bewährte Fragebögen eingesetzt, die ebenfalls Konstrukte psychischer Belastung bei Krebspatienten messen. Das DT ist ein Ultrakurzscreening. Seine einfache Handhabung in Durchführung und Auswertung begünstigte, dass es sich als Screeningverfahren im Bereich der Psychoonkologie in der klinischen Praxis etabliert hat. Die Autoren geben für die deutsche Version einen Cut-off-Wert von ≥5 an, oberhalb dessen eine psychoonkologische Beratung empfohlen wird [14].

Die PO-Bado ist eine Fremdeinschätzungsskala, welche somatische und psychische Belastungen mit vier bzw. acht Items auf einer fünfstufigen Likert-Skala erfasst [8]. Die diagnostische Differenzierung konnte durch signifikante Belastungsunterschiede zwischen verschiedenen Gruppen von Tumorpatienten gezeigt werden. Zur Auswertung klinischer Studien empfehlen die Autoren die Bildung von Mittelwerten.

### Statistik

Die erhobenen Daten wurden mittels SPSS, Version 23.0, ausgewertet. Zur Festlegung des Cut-off-Werts wurden ROC(„receiver operating characteristic“)-Kurven berechnet. Als Maß für die Güte (Diskriminationsfähigkeit) werden die AUC(„area under the ROC curve“)-Werte herangezogen. Zur Überprüfung der Validität des DST wurden etablierte Kennwerte bestimmt (Sensitivität, Spezifität, positive und negative prädiktive Validität sowie Youden-Index) [25]. Weiterhin wurden Skaleninterkorrelationen bestimmt.

## Ergebnisse

### Deskriptive Statistik

Für das DST wurde ein statistisch optimaler Cut-off-Wert von 34 errechnet, mit dem 60 % Patienten als belastet identifiziert werden (*M* = 37,23; *SD* = 10,01; Range 20–68). Mittels eines Cut-off-Werts von 28, der zugunsten einer für ein Screening wesentlicheren höheren Sensitivität empfohlen wird, werden 82 % der Patienten als belastet identifiziert. Für das DT wird der empfohlene Cut-off-Wert verwendet, mit dem 60 % der Patienten als belastet identifiziert werden (*M* = 6,30; *SD* = 2,93; Modalwert 6,0; Range 0–10) [14]. Mithilfe des für die Einzelauswertung genutzten Schwellenwertkriteriums der PO-Bado wurden 59 % der Patienten als belastet identifiziert. Davon abweichend ergeben sich in der Betrachtung der Skala psychische Belastung der PO-Bado 67 % der Patienten, deren Belastung als hoch eingestuft wird (*M* = 12,86; *SD* = 5,21) Auf der Grundlage der Skala somatische Beschwerden wurden 71 % der Patienten als belastet identifiziert (*M* = 8,52; *SD* = 4,06) [7].

### Skaleninterkorrelationen

Aufgrund signifikanter Korrelationen des DST mit DT und den Subskalen des PO-Bado (*r* = 0,39 bis *r* = 0,66; *p*-Werte ≤0,001) kann zuverlässig von einer konvergenten Validität des DST ausgegangen werden. Das Konstrukt des DST entspricht also grundsätzlich denen etablierter Verfahren. Die teils schwachen Skaleninterkorrelationen sind mit der inhaltlichen Weiterentwicklung des DST gegenüber dem DT und der PO-Bado begründet und erwartungskonform.

Für das Schwellenwertkriterium der PO-Bado, das hier als kategoriale Variable (belastet/nicht belastet identifiziert) festgelegt wurde, wurde mithilfe eines festgelegten Cut-off-Werts des DST von 34, aufgrund fehlender Voraussetzungen parametrischer Tests, Cramers V bestimmt. Es zeigt sich ein kleiner mittlerer signifikanter Zusammenhang (Cramers V = 0,29; *p* *<* 0,001; Tab. 2).

**Table 2:** 

	DST
DT	0,39
PO-Bado psychische Belastungen	0,66
PO-Bado somatische Belastungen	0,46
PO-Bado Schwellenwert	0,29^a^

*DT* Distress-Thermometers, *PO-Bado* Psychoonkologischen Basisdokumentation, *DST* Düsseldorfer Screeningtool

^a^Hier wurde Cramers V berechnet

### Sensitivität und Spezifität des Düsseldorfer Screeningtools

Die Tab. 3 zeigt die Ergebnisse der ROC-Analysen für verschiedene aus der Literatur übernommene Cut-off-Werte des DT (Cut-off = 4, 5, 6). Legt man den in Deutschland am häufigsten verwendeten Cut-off-Wert für das DT von 5 zugrunde, liegt die Diskriminationsfähigkeit des DST bei 0,66 (AUC). Die Tab. 4 gibt einen Überblick über die Ergebnisse der ROC-Analysen mithilfe der verschiedenen Auswertungsmöglichkeiten der PO-Bado-Skalen. Hier liegt die Diskriminationsfähigkeit bei 0,72 bis 0,80 (AUC). Die Sensitivitätswerte und Spezifitätswerte werden zur besseren Veranschaulichung nur für die Cut-off-Werte dargestellt, deren Youden-Index in einem moderaten bis guten Bereich liegt.

**Table 3:** 

DT							
	DST Cut-off	Sensitivität (%)	Spezifität (%)	AUC	*p*	KI	YoudenIndex
Cutoff 4	28	88,6	32,4	0,71	<0,001	0,680,82	0,21
	29	84,1	44,1				0,28
	30	80,7	47,1				0,28
	31	76,1	50,0				0,26
	32	72,7	58,8				0,32
	33	72,7	64,7				0,37
	34	71,6	70,1				0,42
Cutoff 5	28	88,2	26,1	0,66	<0,001	0,560,76	0,14
	29	84,2	37,0				0,21
	30	80,3	39,1				0,19
	31	75,0	41,3				0,16
	32	72,4	50,0				0,23
	33	72,4	54,3				0,26
	34	71,1	58,7				0,29
	35	63,2	60,9				0,24
	36	60,5	67,4				0,28
Cutoff 6	30	84,5	37,5	0,64	0,009	0,530,74	0,22
	31	77,6	39,1				0,17
	32	74,4	45,3				0,20
	33	74,1	48,4				0,23
	34	72,4	51,5				0,24

*DT* Distress-Thermometers, *DST* Düsseldorfer Screeningtool, *AUC* „area under curve“, *KI* Konfidenzintervall

**Table 4:** 

PO-Bado							
	DST Cut-off	Sensitivität (%)	Spezifität (%)	AUC	*p*	KI	YoudenIndex
Psychische Belastungen	28	92,9	43,6	0,80	<0,001	0,72–0,88	0,36
	29	86,9	51,3				0,38
	30	83,3	53,8				0,37
	31	79,8	59,0				0,38
	32	76,2	66,7				0,43
	33	75,0	66,7				0,42
	34	72,6	69,7				0,42
Somatische Belastungen	28	89,9	41,2	0,79	<0,001	0,71–0,87	0,31
	29	86,5	55,9				0,42
	30	83,1	58,8				0,42
	31	78,7	61,8				0,40
	32	71,9	61,8				0,34
	33	71,9	64,7				0,36
	34	69,7	67,6				0,37
Schwellenwertkriterium	28	87,8	26,9	0,72	<0,001	0,64–0,81	0,15
	29	83,8	36,5				0,20
	30	79,7	38,4				0,18
	31	75,7	42,3				0,18
	32	73,0	50,0				0,23
	33	73,0	53,8				0,27
	34	71,6	57,6				0,29

*DT* Distress-Thermometers, *PO-Bado* Psychoonkologischen Basisdokumentation, *DST* Düsseldorfer Screeningtool, *AUC* „area under curve“, *KI* Konfidenzintervall

Bei einer Festlegung des Cut-off-Werts für das DST von ≥34 liegt bei nahezu allen Vergleichsanalysen unter gleichzeitiger Berücksichtigung von Spezifität und Sensitivität das beste diagnostische Entscheidungsrational zugrunde. Hier zeigt sich als höchster Sensitivitätswert 72,6 % bei gleichzeitiger Spezifität von 69,7 %. Zugunsten des für ein Screeningverfahren wichtigeren Merkmals der Sensitivität ergeben sich mögliche Cut-off-Werte von ≥30 sowie ≥28. Hier konnten im Vergleich zur Skala somatische Belastung der PO-Bado eine Sensitivität von 86,5 % bei gleichzeitiger Spezifität von 55,9 % sowie im Vergleich zur Skala psychische Belastung der PO-Bado eine Sensitivität von 92,9 % bei gleichzeitiger Spezifität von 43,6 % ermittelt werden. Für diese Cut-off-Werte wurden zusätzlich die Werte für die positive prädiktive Power (PPP), die negative prädiktive Power (NPP) sowie die „cross-product ratio“ (CPR) berechnet (Tab. 5). Zur Berechnung der prädiktiven Power wurde aus der Literatur eine Prävalenz für psychische Belastung von 40 % für diese Stichprobe abgeleitet [16, 20].

**Table 5:** 

Cut-off DST	CPR	PPP	NPP
28	10,11	52,3	90,1
30	8,12	56,7	86,1
34	5,31	59,2	78,5

*DST* Düsseldorfer Screeningtool, *CPR* „cross-product ratio“, *PPP* positive prädiktive Power, *NPP* negative prädiktive Power

Vergleicht man die berechneten Werte des DST mit den Ergebnissen des DT an der gleichen Stichprobe, so sind die Gütekriterien des DST als günstiger und in Teilen als deutlich günstiger einzustufen (Tab. 6).

**Table 6:** 

DT und PO-Bado										
	DT Cut-off	Sensitivität (%)	Spezifität (%)	AUC	*p*	KI	Youden-Index	CPR	PPP	NPP
Psychische Belastungen	4	89,2	36,1	0,74	0,000	0,65–0,84	0,13	4,67	48,20	83,37
	5	81,9	50,0				0,12	4,52	52,20	80,56
	6	73,3	61,1				0,12	4,31	55,68	77,44
Somatische Belastungen	4	86,2	31,25	0,67	0,005	0,56–0,78	0,18	2,84	45,53	77,26
	5	79,3	46,9				0,26	3,38	49,89	77,27
	6	70,1	56,2				0,26	3,01	51,62	73,82
Schwellenwertkriterium	4	93,1	36,0	0,75	0,000	0,67–0,84	0,29	7,59	49,23	88,67
	5	84,7	46,0				0,31	4,72	51,12	81,85
	6	79,2	62,0				0,41	6,21	58,15	81,72

*DT* Distress-Thermometers, *PO-Bado* Psychoonkologischen Basisdokumentation, *AUC* „area under curve“, *KI* Konfidenzintervall, *CPR* „cross-product ratio“, *PPP* positive prädiktive Power, *NPP* negative prädiktive Power

### Subjektiver Unterstützungsbedarf

Dass sie sich bei der Verarbeitung ihrer Erkrankung Unterstützung wünschen, geben 68% der Patienten an. Kreuztabellen zeigen, dass 75 % derjenigen, die Unterstützungsbedarf äußerten, auch durch das DST als unterstützungsbedürftig eingestuft werden. Die Einstufung der Unterstützungsbedürftigkeit erfolgte durch die Skala 1 des DST. Mittels DT und PO-Bado konnten 74 % bzw. 75 % Deckungsgleichheit bzgl. einer Aussage zum Unterstützungsbedarf erzielt werden. Bezogen auf einen Cut-off-Wert von 28 des DST stimmen Patientenauskunft und Einstufung durch das Screening in 87 % der Fälle überein.

## Diskussion

Das DST ist ein Screeningverfahren, dass die psychische Belastung infolge einer Krebserkrankung unter Berücksichtigung stabiler Traits, des Krankheitsverarbeitungsstils sowie der sozialen Unterstützung der Patienten valide und reliabel misst. Erstmalig berücksichtigt ein Screening neben akuten Symptomen auch Ressourcen sowie interne Risikofaktoren des Patienten. Damit gilt es als ein Verfahren, welches die diagnostischen Anforderungen aus psychoonkologischen Forschungsergebnissen auch inhaltlich berücksichtigt [10]. Darüber hinaus ermöglicht das DST eine schnelle und zuverlässige Beurteilung des objektiven und subjektiven Unterstützungsbedarfs, ohne nachträglich Informationen am Patienten erheben zu müssen.

Die in dieser Stichprobe untersuchten Patienten zeigen erwartungsgemäß höhere Belastungswerte als Patienten mit anderen Krebserkrankungen. Mittels der drei hier verwendeten Screeningverfahren wurden zwischen 59 und 81 % der Patienten aufgrund erhöhter psychischer Belastung als behandlungsbedürftig identifiziert. Damit zeigt sich in dieser Stichprobe eine deutlich höhere Anzahl an belasteten Patienten als in Vergleichsstudien mit einer ähnlichen Klientel [16, 20]. Gründe dafür könnten im Zeitpunkt der Erhebung gesehen werden. Alle Patienten wurden im Rahmen der Primärtherapie häufig sehr kurz nach der Diagnosestellung befragt. Kruijver et al. weisen in ihrer Übersichtsstudie darauf hin, dass die Patienten in dieser frühen Phase möglicherweise noch nicht bereit sind, sich neben der Beschäftigung mit der Diagnose Krebs mit weiteren Belastungen auseinanderzusetzen. Gleichzeitig zeigen sie jedoch auf, dass insbesondere nach Abschluss erster tumorentfernender Maßnahmen ein psychoonkologisches Screening den Patienten ein positives Gefühl der ganzheitlichen Fürsorge vermittelt [11].

Insgesamt weist das DST im Vergleich zu den anderen Verfahren eine gute Diskriminationsfähigkeit auf. Bei AUC-Werten von 0,72 bis 0,80 ist davon auszugehen, dass das neue Screening einen Mehrwert bei der Erfassung psychischer Belastung leistet.

Ein Cut-off-Wert von 34 erzeugt, sowohl unter Nutzung der PO-Bado als auch des DT als diagnostischem Äquivalent, bei allen Cut-off-Werten eine bestmögliche Ausgewogenheit von Sensitivität und Spezifität. Bei höherer Gewichtung der Sensitivität erscheint, auch unter der Berücksichtigung der „cross-product ratio“ und der positiven und negativen prädiktiven Power, ein Cut-off-Wert von 28 als in der Praxis angemessener. Hier konnte im Vergleich zur PO-Bado eine Sensitivität von 92,9 % bei gleichzeitiger Spezifität von 43,6 % ermittelt werden.

Im Vergleich zu einer Nutzung der PO-Bado stellt das DST eine deutlich ressourcenschonendere Variante des Screenings dar. Zum einen wird hier aufgrund der Selbsteinschätzung durch den Patienten kein zusätzliches medizinisches oder psychologisches Personal benötigt. Zum anderen ist die Auswertung mittels Summenbildung und Cut-off-Wert deutlich einfacher und zeitsparender als die Bildung von Schwellenwerten.

Im Vergleich der Gütekriterien zeigt sich eine ähnliche Diskriminationsfähigkeit des DST und des DT. Der Mehrwert des DST im Vergleich zum DT ergibt sich demnach vor allem durch die Möglichkeit, Risikofaktoren und persönliche Ressourcen innerhalb des initialen Screenings zu erkennen und gezielt Interventionsmöglichkeiten anzubieten sowie den objektiven und subjektiven Unterstützungsbedarf patientenzentriert zu beurteilen. Die Erkenntnis über patienteneigene Ressourcen kann dann dazu führen, diese verstärkt in den Heilungsprozess einzubeziehen. Der Entscheidungsprozess bei der Behandlungsstrategie wird durch das DST somit qualitativ unterstützt.

Die separate Erhebung des Wunsches nach Unterstützung, die in das DST mittels einer Frage zum grundsätzlichen Bedarf und drei weiteren Fragen zur inhaltlichen Spezifizierung aufgenommen wurde, nimmt die Forderung auf, neben einem objektiven Testwert auch das subjektive Empfinden der Patienten zu berücksichtigen. In dieser Stichprobe gaben 75 % der als belastet identifizierten Patienten auch von sich aus den Wunsch nach Unterstützung an. Damit liegen die hier festgestellten Zusammenhänge etwas höher als in anderen Studien [16, 20]. Die zusätzlichen Items zum Unterstützungsbedarf können zusammen mit den Items zur Messung positiver Ressourcen genutzt werden, um schnell eine für den Patienten passende Möglichkeit der Intervention unter Berücksichtigung seiner persönlichen Vorstellungen und Stärken bereitzustellen. An dieser Stelle bietet das DST trotz seiner kurzen Bearbeitungszeit ein Informationsinkrement im Vergleich zu bestehenden Screenings, das praktische Implikationen für die individuelle Therapieplanung aufzeigt. Die Praktikabilität des DST im klinischen Alltag muss in zukünftigen Studien untersucht werden.

## Limitationen

- Die Wahl der als Zustandsvariable definierten Verfahren ist nicht optimal. Rund ein Fünftel der durch die DT als belastet identifizierten Patienten sind objektiv nicht belastet [15]. Es ist also fraglich, ob das DST die Prävalenz psychischer Belastung daher überschätzt. In zukünftigen Untersuchungen sollte die konvergente Validität daher durch die Nutzung klinischer Fragebögen überprüft werden.
- Aufgrund der inhaltlich verschiedenen Konstruktion des DST ist davon auszugehen, dass die durch den DST gemessene Belastung nicht deckungsgleich mit den etablierten Verfahren ist. Weitergehende Studien zur Bestimmung der diskriminanten sowie prognostischen Validität sind daher notwendig, um den Wert des DST substanziell zu belegen.
- Die hier gewählten Cut-off Werte sind nicht generalisierbar. Die Normierung für verschiedene Krebserkrankungen und Kohorten ist notwendig.
- Die hier untersuchte Stichprobe befand sich in der Primärtherapie. Eine Anwendung in späteren Behandlungsphasen ist aufgrund der genutzten Items denkbar. Derzeit läuft im Universitätsklinikum Düsseldorf eine entsprechende Langzeitstudie, um dies zu validieren.

## Ausblick

Die Ergebnisse dieser Studie zeigen, dass zur Ableitung eines Behandlungsbedarfs einzig Fragen zur akuten Belastungssituation nicht ausreichen. Zum effektiven Einsatz psychoonkologischer Kapazitäten ist es wesentlich, zu erfassen, wie die Belastung infolge der Krebserkrankung zum Zeitpunkt der Erhebung als auch in Zukunft beschaffen ist. Die Erfassung vorhandener Ressourcen und Risikofaktoren bietet eine zuverlässige Möglichkeit, psychische Belastung zu identifizieren. Dieser in der Psychoonkologie neue Ansatz bietet einen erkenntniskonformen und ökonomischen Ansatz zum Screening.

## Fazit für die Praxis

- Die Fokussierung auf zeitlich stabile Risikofaktoren und Ressourcen eines Patienten ist zielführend bei der Erfragung der psychischen Belastung von Krebspatienten.
- Für Patienten, die aufgrund der hohen Augenscheinvalidität belastungsorientierter Fragen dazu neigen, ihre Belastung zu hoch oder zu niedrig einzuschätzen, ist die Belastungserfassung mittels DST eine gelungene Alternative.

## Supplementary Information





