# Supplementary material for: Validation of the Düsseldorfer screening tool: a trait-based approach to assess psychological distress of cancer patients
Source: HNO. 2020 Dec 18;69(11):899–906. [Article in German] doi: 10.1007/s00106-020-00980-4 (PMC8545723; doi:10.1007/s00106-020-00980-4)
Supplement: Supplementary file 1 [file 106_2020_980_MOESM1_ESM.pdf]

Stichprobenbeschreibung: Geschlecht, Familienstand und Krankheitsbild

	**Anteil der Patienten in %**
**Geschlecht**	
weiblich	40 %
männlich	60 %
**Familienstand**	
ledig	11 %
in einer Beziehung	7%
verheiratet	56 %
verwitwet	16%
geschieden	10%
**Tumorlokalisation**	
Lippe	5%
Zungengrund	2%
Zunge	18%
Zahnfleisch	9%
Mundboden	18%
Gaumen	2%
Sonstige Bereiche des Mundes	8%
Parotis	2%
Oropharynx	2%
mehrere Teilbereiche überlappend	27%
Kopf	5%
Auge	1%
Ohr	1%
