# Supplementary material for: Validation of the Düsseldorfer screening tool: a trait-based approach to assess psychological distress of cancer patients
Source: HNO. 2020 Dec 18;69(11):899–906. [Article in German] doi: 10.1007/s00106-020-00980-4 (PMC8545723; doi:10.1007/s00106-020-00980-4)
Supplement: Supplementary file 2 [file 106_2020_980_MOESM2_ESM.pdf]

Tabelle 1: Skala 1 Ressourcendefizite

Items	M	SD	r_{it}
3. Ich bekomme leicht das Gefühl, in Panik zu geraten.	1.92	.89	.55
4. Ich fühle mich häufig allein gelassen.	1.66	.94	.33
5. Im Umgang mit anderen fühle ich mich unsicher.	1.52	.80	.62
6. Ich fühle mich antriebslos.	1.68	.82	.64
8. Es sind Einschränkungen durch die Erkrankung eingetreten, mit denen ich nur schwer umgehen kann.	2.39	1.06	.68
9. Ich fühle mich ständig gestresst.	1.52	.69	.58
10. Den Verlauf meiner Erkrankung kann ich durch nichts beeinflussen.	2.31	1.01	.47
11. Ich grüble oft über mein Leben nach.	2.50	1.00	.57
12. Ich habe das Gefühl wertlos zu sein.	1.32	.79	.45
15. Nach einem Rückschlag erhole ich mich nur schwer.	2.37	.92	.46
16. Ich bin häufig niedergeschlagen.	1.90	.918	.81
17. Niemand versteht, wie schlecht es mir geht.	1.68	.845	.68
19. Es ist schwierig für mein Umfeld, sich in meine Situation hineinzuversetzen.	2.15	.95	.63
21. Ich habe kein Interesse mehr an Dingen, die ich früher gern gemacht habe.	1.81	.93	.66
22. Ich fühle mich durch meine körperlichen Beschwerden im Alltag eingeschränkt.	2.39	1.06	.65
23. Ich ziehe mich von anderen Menschen zurück, wenn es mir schlecht geht.	2.10	1.08	.56
24. Ich rege mich schnell über andere Menschen auf.	1.98	.89	.49
28. Ich fühle mich im Alltag oft überfordert.	1.89	.89	.78
29. Durch meine Erkrankung bin ich in meinem religiösen Glauben erschüttert.	1.42	.933	.59
30. Ich bin sehr angespannt.	2.16	.89	.66

Anmerkung: M=Mittelwert; SD=Standardabweichung; r_{it}=Itemtrennschärfe

Tabelle 2: Skala 2 positive Ressourcen

**Items**	**M**	**SD**	r_{it}
1. Auch in schwierigen Zeiten erwarte ich normalerweise das Beste.	3.10	.78	.44
2. Ich verfolge meine Ziele energisch.	3.04	.65	.39
6. Ich kämpfe entschlossen gegen meine Erkrankung an.	3.70	.64	.40
13. Wenn andere Probleme haben, bin ich gerne für sie da.	3.30	.75	.30
14. Ich genieße jeden Moment, in dem es mir gut geht.	3.62	.62	.68
18. Meine Zukunft sehe ich immer optimistisch.	3.30	.88	.63
20. Ich kann durch mein eigenes Verhalten etwas an meinem Befinden ändern.	3.00	.82	.32
25. Ich war mit meinem bisherigen Leben im Allgemeinen zufrieden.	3.51	.58	.59
26. Wenn es mir nicht gut, ist immer jemand für mich da.	3.42	.67	.40
27. Ich habe mir für mein Leben noch viel vorgenommen.	3.09	.95	.44

Anmerkung: M=Mittelwert; SD=Standardabweichung; r_{it} =Itemtrennschärfe

Tabelle 3: subjektiver Unterstützungsbedarf

**Item**	**M**	**SD**	r_{it}
31. Ich brauche keine Hilfe, um meine Erkrankung zu verarbeiten.	2.06	.97	.05
32. Ich wünsche mir einen Termin in einer Beratungsstelle.	1.60	.93	.45
33. Ich wünsche mir Anschluss an eine Selbsthilfegruppe.	1.47	.65	.51
34. Ich wünsche mir Unterstützung durch einen Pfarrer.	1.24	.98	.14

Anmerkung: M=Mittelwert; SD=Standardabweichung; r_{it} =Itemtrennschärfe
